# Minimal Developmental Computation: A Causal Network Approach to Understand Morphogenetic Pattern Formation

**DOI:** 10.3390/e24010107

**Published:** 2022-01-10

**Authors:** Santosh Manicka, Michael Levin

**Affiliations:** Allen Discovery Center, Tufts University, Medford, MA 02155, USA; santosh.manicka@tufts.edu

**Keywords:** biological computation, developmental patterning, distributed information processing, collective phenomena, causal information flow, biological circuits, artificial embryogeny

## Abstract

What information-processing strategies and general principles are sufficient to enable self-organized morphogenesis in embryogenesis and regeneration? We designed and analyzed a minimal model of self-scaling axial patterning consisting of a cellular network that develops activity patterns within implicitly set bounds. The properties of the cells are determined by internal ‘genetic’ networks with an architecture shared across all cells. We used machine-learning to identify models that enable this virtual mini-embryo to pattern a typical axial gradient while simultaneously sensing the set boundaries within which to develop it from homogeneous conditions—a setting that captures the essence of early embryogenesis. Interestingly, the model revealed several features (such as planar polarity and regenerative re-scaling capacity) for which it was not directly selected, showing how these common biological design principles can emerge as a consequence of simple patterning modes. A novel “causal network” analysis of the best model furthermore revealed that the originally symmetric model dynamically integrates into intercellular causal networks characterized by broken-symmetry, long-range influence and modularity, offering an interpretable macroscale-circuit-based explanation for phenotypic patterning. This work shows how computation could occur in biological development and how machine learning approaches can generate hypotheses and deepen our understanding of how featureless tissues might develop sophisticated patterns—an essential step towards predictive control of morphogenesis in regenerative medicine or synthetic bioengineering contexts. The tools developed here also have the potential to benefit machine learning via new forms of backpropagation and by leveraging the novel distributed self-representation mechanisms to improve robustness and generalization.

## 1. Introduction

How does a developing embryo self-organize into a patterned structure arranging the various differentiated morphological features using input information from its own cells [1,2]? In essence, how does an embryo compute its own pattern [3,4,5,6,7,8,9]? Morphogenesis, whether embryonic or regenerative, is an intriguing paradigm for computer science, in addition to biology and biomedicine, because it provides proof-of-principles of a dynamic, strongly embodied computational architecture [10,11,12]. The results of its computations alter the architecture and structure of the computing medium in real-time. Morphogenesis is extremely robust (reviewed in [13]), partly because it has evolved to maintain reliable signaling and morphological decision-making while it itself is actively remodeling. While progress in molecular genetics has made huge strides in uncovering the mechanisms *necessary* for morphogenesis, there are still significant gaps in a mature understanding of the strategies and meso-scale design principles *sufficient* to enable the right combination of reliability and plasticity [14,15,16]. How do cellular collectives make decisions with respect to signaling and cell behavior that reliably result in the correct species-specific target morphology?

To answer such questions, various conceptual and computational models have been developed [17,18,19,20,21,22,23,24]. Lewis Wolpert, a pioneer in this field, introduced the concept of “positional information”, where a morphogen gradient develops along an axis that the cells could use to decode their relative positions and make morphogenetic decisions in embryogenesis or regeneration [25]. He used the famed metaphor of the ‘French-flag’ to characterize a gradient-like pattern and later proposed models for how such patterns could be developed, sustained and even regenerated [25,26,27]. Pattern formation is a dynamical and multifaceted phenomenon that is not restricted to embryogenesis. For instance, a variety of biological systems possess capabilities of regenerative patterning, that is, adult forms can regenerate full patterns from sub-regions when severed [28]. To understand patterning in its various forms, therefore, several conceptual and mathematical models have been developed since Wolpert’s work. A few notable concepts include “organizers”, morphogenetic fields, epimorphosis, morphallaxis and intercalation [20,29,30]. The concept of intercalation, for instance, explains how an arbitrary piece of a planarian regenerates the whole worm; the anterior and posterior poles of the stump have non-adjacent “positional values” relative to their neighbors, the continuity of which is then gradually restored leading to the formation of the full original pattern. Most notable mathematical models of patterning are based on either simple diffusion or the more complicated reaction-diffusion mechanisms [19,21,22,24], with some exceptions like the ”clock and wavefront model” [31]. A model introduced by Alan Turing, known as “Turing patterns”, for instance, is based on a mechanism of “short-range activation and long-range inhibition” driven by inherent dynamical instabilities [32]. A variety of computational models that view cells as discrete entities (“agents”) containing memory and performing actions or use artificial neural networks in the background for morphogenetic decision-making have been attempted as well [33,34,35,36,37,38]. Even though these models explain certain aspects of patterning in concise and intuitive ways, they do not reveal the full underlying dynamical richness of this nonlinear phenomenon. For instance, a typical analysis of Turing patterns predicts the size and wavelength of the emergent patterns using mathematical expressions involving the ratio of the activator and inhibitor diffusion coefficients obtained by linearizing the model. Even though this approach is useful, it still lacks the full picture of a *nonlinear computational* account of patterning that moreover is also interpretable. Such an account may be invaluable for systematically addressing the inverse patterning problem where one wishes to modify the underlying regulatory systems to achieve desired patterning outcomes [13,39].

One of the central challenges with respect to models is thus an understanding of the high-level information-processing principles they leverage towards the patterning outcomes. For instance, the “balancing model” proposed by Wolpert represents cells as point-entities that make linear measurements such as computing simple averages. As helpful as the model is in hypothesizing high-level principles, they do not elucidate the underlying biological complexity. Models based on reaction-diffusion or artificial neural networks, on the other hand, capture some of the biological complexity but suffer from the drawback that it is difficult to elucidate the underlying nonlinear causal relationships using conventional analysis techniques [40,41].

Information theory offers tools [42,43,44] to overcome some of the above-mentioned barriers by inferring the complex causal relationships among the components of a system from timeseries data using a probabilistic perspective [45], although not without challenges [46]. In the same way, dynamical systems offer tools to infer “dynamic causal models” (DCM) [47] that elucidate the causal relationships underlying a dynamical phenomenon from a deterministic (non-probabilistic) perspective [45]. These efforts to explicate the information-processing mechanisms of complex dynamical models have given birth to a new field dedicated to solving the problem of “interpretability” [48]. As a representative example, the method of “saliency map” elucidates the parts of an input image that an output neuron of an image-recognizing ANN is most sensitive to by computing the mathematical derivative of the output with respect to the input [49,50], thereby revealing the character of that neuron in a human-interpretable way. Although saliency map and its variants [50] have been used to characterize the high-level information-processing principles of ANNs, they have not been used to similarly analyze non-neural biological models, to the best of our knowledge. With the advent of differentiable programming [51], the time is ripe for the extension of those methods to non-neural recurrent dynamical models. In summary, the limitations of conventional analysis methods’ inability to reveal effective information-processing principles can now be surpassed by extending the techniques developed for interpreting ANNs. Besides, we can now develop methods to more closely link computation in systems whose outputs modify the computing medium and use machine learning to discover morphogenetic control strategies that serve as hypotheses for developmental biology or design policies for synthetic bioengineering.

The goal of this paper is thus two-fold: (1) to design a minimal nonlinear dynamical model of generic pattern formation using plausible biological priors; and (2) to characterize the high-level organizational principles that the model employs by applying analysis methods that are extensions of conventional methods used for ANNs.

## 2. Model and Methods

**Figure 1:**
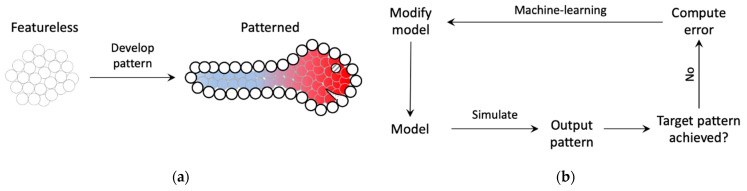
Schematic of the modeled biological phenomenon and how machine-learning is used to design the model. (**a**) During early embryogenesis, a relatively featureless embryo develops distinct axial patterns along with a distinct outer layer known as the epidermis (figure of larva was inspired by [1] (p. 11)). The distinct colors represent the origin of the differentiation of the embryo into distinct morphological features. The cells on the boundary (thick empty circles) represent the epidermis. (**b**) A model with unknown parameters is trained using machine-learning, that uses gradient-descent-like methods to ‘backpropagate’ the error between the observed and target patterns to the model parameters, to produce the desired pattern.

We designed a minimal model of bounded axial patterning—a phenomenon that commonly occurs during embryogenesis and regeneration where a featureless tissue develops an epidermis (boundary) and an axis within it (e.g., anterior-posterior, dorsal-ventral, etc.) along which distinct morphological features later appear (Figure 1). Rather than simulating any one species’ morphogenesis, we seek to understand axial polarity, a generic mechanism used throughout biology [52,53,54,55,56,57,58,59,60].

**Figure 2:**
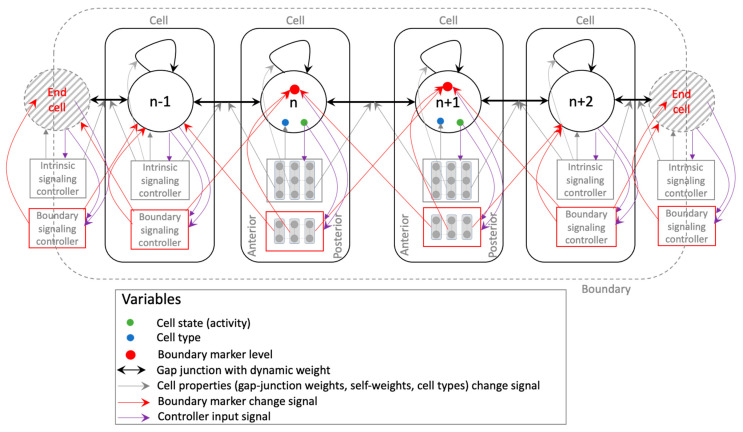
Schematic of the model of axial pattern development. The model comprises of a finite linear chain of cells (a total of 12 cells comprise the model used in this work). We hypothesize that the following elements will be sufficient to implement emergent axial patterning. Every cell has two kinds of signaling networks, one that determines the properties of the cell, namely the cell type and the gap-junction weights, and the other that signals to the cell the extent to which it lies at the boundary. These two factors are expected to act in concert in simultaneously detecting the boundaries and developing a gradient-like phenotypic pattern within the detected boundaries where the pattern would be expected to taper off. The intrinsic controller is a 3 × 3 lattice (totaling 9 nodes), and the boundary controller is a 2 × 3 lattice (totaling 6 nodes), depicted as part of the representative cells n and (n+1) in the middle. As the connections indicate, the anterior and posterior (1st and 3rd) columns of the intrinsic controller influence the anterior and posterior gap-junction weights respectively, and the central column influences the cell’s self-weight. In the same way, the most anterior and posterior (1st and 3rd) columns of the boundary controller signal to the anterior and posterior cells, respectively. Finally, in each cell, the cell state influences all the nodes of either controller, the cell type is influenced by all of the intrinsic controller nodes, and all of the boundary controller nodes are influenced by the cell’s own boundary-marker level.

**Figure 3:**
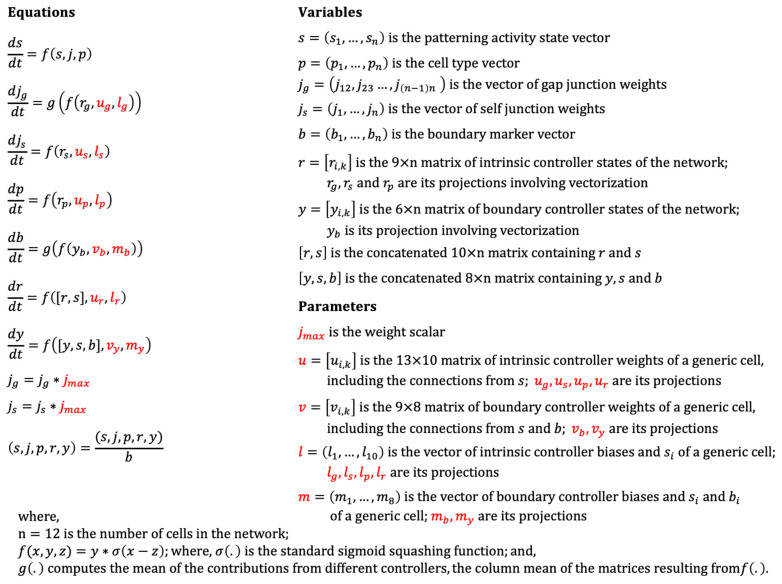
Formal definition of the model. All variables and parameters are continuous in that they can in principle assume any value on the real number line. The parameters (red) are trained and thus fixed during simulations. All variables are scaled by the boundary marker level b, except itself, representing its dampening effect (akin to a time constant)—the greater the value of b (the greater the cell tends to be a boundary cell), the more dampened its overall activity, including the patterning state, cell properties and internal controller states, is. Subscripted parameters and variables denote unique projections of the corresponding parameters and variables that can be inferred from the connectivity diagram shown in Figure 2. In cases where the value of a variable is determined by inputs from multiple controllers, such as the gap-junctions and the boundary-markers, the averaging operator g() computes the means of those contributions. The case of gap-junction weight updates exemplifies these concepts. The weight of every gap-junction $j_{\left(n-1\right)n}$ is determined by the intrinsic controllers of cells (i−1) and i, specifically by the posterior column in the controller of the anterior cell and the anterior column of the posterior cell. Accordingly, the dimension of $r_{g}$ would be 3×2×(n−1), where the dimensions 3×2 represents the three nodes each of the two controller columns. This $r_{g}$ would then be partially vectorized, with the two columns contributing to each gap-junction concatenated into one, yielding a matrix with dimensions 6×(n−1). In the same way, the dimension of $u_{g}$ would be 2×6, representing the two gap-junctions each contributed to by one column of the generic cell’s intrinsic controller totaling six nodes (three nodes per column). Thus, the multiplication of $u_{g}$ with $r_{g}$ yields a matrix of dimensions 2×(n−1). Finally, the averaging operator g() computes the column-wise mean of the previous matrix resulting in a 1×(n−1) vector of updates to all the gap-junctions.

The functional output sought in this model is to develop an axial pattern of positional information at the same time marking the boundary (epidermis) within which to develop it (Figure 1). Even though the model is simplified, it captures certain biological priors that orchestrate embryogenesis, such as intra-cellular network control (e.g., genetic, biochemical signaling) with features supporting the implementation of planar cell polarity, local intercellular connectivity, and bounded development. The following are the main features of the model (Figure 2). It consists of a bounded (finite) linear chain of cells, each characterized by a ‘cell type’, and connected by edges representing gap-junctions [61] that allow them to communicate physiologically. Every cell, furthermore, contains a self-regulating connection representing autocrine signaling in biological cells that gives them a capacity to regulate themselves [62] (p. 238). The cell type, along with the gap junctions and self-regulating connections, dictates the cell’s behavior (response to inputs). The dynamical state (output) of a cell is referred to as its “activity level” that is steered by its interactions with the neighboring (input) cells’ activity levels; this represents the cell’s emergent positional information and identity within the tissue. The connection weights and the cell types are themselves dynamic variables whose properties are determined by an “intrinsic signaling controller” that each cell possesses internally with an architecture shared across all cells, representing the generic signaling networks that biological cells possess (e.g., genetic networks, biochemical signaling networks, etc.). The dynamic nature of the gap-junctions represents the dynamically changing permeability of their biological counterparts due to voltage-gating, for example [63]. Furthermore, since a gap-junction connects two adjacent cells, its dynamic weight is jointly determined by the cells’ intrinsic controllers. Every cell also possesses a “boundary-marker” state, a dynamical variable representing the extent to which the cell is deemed to lie at the boundary—the higher the value of the state, the larger is the cell deemed to be at the boundary. Like the activity state, the boundary-marker state of a cell depends on the boundary-marker states of the neighboring cells as well as itself. These boundary-marking signals are generated by a second intracellular controller known as the “boundary signaling controller”. The function of the boundary-marker is to dampen all the activities of a cell to a degree proportional to the level of the boundary-marker (Figure 3)—the more a cell tends to be a boundary cell, the more dampened its overall activity is, including the patterning state, cell properties and the internal controller states. This feature represents the biological setting where the embryonic epidermis is relatively less active compared to the inner cells. In this regard, the boundary-marker represents the antagonistic version of “growth factors” that biological cells signal to neighboring cells to divide [64]. Another important feature of the model is that the intrinsic controller is designed to support the implementation of planar cell polarity (PCP) that gives the cell a sense of polarity, a feature that is known to play an important role in the development of organism-level axial polarity [65,66]. In the model, specifically, the anterior (left) column of the intrinsic controller influences the anterior gap-junction weight, whereas the posterior (right) column influences the posterior gap-junction weight (Figure 2). This arrangement allows machine-learning to assign distinct modes of behavior to the two polar columns, thereby effectively functioning as PCP (described in more detail below). Lastly, the fact that every cell in the model possesses two separate internal controllers tasked with unique functionalities represents the biological analogue of multiple signaling networks working in concert inside a cell, representing for example the biochemical and bioelectrical control systems [67].

In summary, while the cells communicate over the gap-junctions to form the target pattern within a boundary, their internal signaling networks help build the tissue-level intercellular network and the network-boundary themselves.

The formal definition of the model is depicted in Figure 3. The model is defined by a set of recurrent ordinary differential equations (ODE) that describe how the variables of the model, namely, the activity states (s), cell types (p), gap-junction weights $\left(j_{g}\right)$, self-weights ($j_{s})$, boundary-marker states (b), intrinsic controller states (r) and the boundary-marker states (y) interact with each other. These variables are ultimately governed by a set of parameters (shown in red in Figure 3) that are trained by machine-learning. We used a specific machine-learning method called backpropagation through time (BPTT) to identify appropriate model parameters that solve the patterning problem. This system models the following aspects of biology. The machine learning loop represents the role of the evolutionary process, which selects for effective morphogenesis by providing cells with a genome encoding the correctly parametrized regulatory network. As occurs in biological evolution, this parameterization process is external to the lifetime of an individual creature and its genomically specified network. In turn, the specific networks produced by the training algorithm represent the results of genomes—the operation of individuals during the morphogenesis of their embryonic development.

The specific problem that the model learned to solve comprises of two main features (details below): (1) a network activity state pattern that is shaped like a simple gradient, with positive values at the anterior and negative values at the posterior and tapering off (goes to zero) at either pole; and (2) a boundary marker pattern where the boundary cells have double the value compared to the cells in between (a value of 2 was set as the target for the stop cells and 1 for the rest). These target patterns were meant to serve as idealized axial polarity and boundary marker patterns to encourage the model to develop patterns that approximately match them.

The method of BPTT broadly involves instantiating a model with random parameters chosen from a specific range, simulating the model for a certain number of steps, then calculating the loss as the difference in the observed network activity and boundary marker patterns with the corresponding set targets and finally backpropagating the loss back to the parameters. The loss function involved a simple mean squared error (MSE) between the observed and the target patterns with equal weights assigned to the network-activity and the boundary-marker patterns. This process was repeated (over several thousands of iterations) until a model with a satisfactory performance was obtained. The parameters u,v,l and m were initialized in the interval [−1, 1], and the parameter $j_{max}$ in the interval [1, 2]. The initial conditions of all the variables were set to 0 during the training. We utilized the software package *Pytorch* [68], that employs automatic differentiation techniques, to implement BPTT.

We sought to explore potential information-processing strategies and general principles that biological systems may employ for the purpose of developmental patterning. To that end, we answer the following questions in this paper: (1) can the model be trained to solve the patterning problem? and (2) how does a successful model work, specifically, how is the information about the target network activity pattern organized in the intrinsic controllers? To answer the second question, we formulated a measure of causal influence (CI) to quantify a graded measure of information—the more a variable x is said to causally influence a variable y in the context of the model’s full state, the more x is deemed to contain information about y in that context. Specifically, we quantify the amount of information contained by variable x(t) about variable y(t+τ) via a measure of CI at timescale τ, defined as ∂y(t+τ)/∂x(t), evaluated in the context of the overall model state at t; the higher the absolute value of this derivative is, the greater is the causal influence. We ascribe the causality of this measure to the necessity and sufficiency of x(t) to impart a change in y(t+τ) in a local sense; a small change in x(t) in one direction will cause a change in y(t+τ) in precisely one direction depending on the sign of the derivative. Moreover, CI is directional and asymmetric by definition; it flows from the source x at time t to the target y at time (t+τ).

In the rest of the paper, we use this formulation of multi-timescale causal influence to quantify the amount of information about patterning activity state s(t+τ) contained in the intrinsic controller states r(t), defined as the following Jacobian tensor evaluated at the full network state at t:

(1) $$J_{s}\left(\tau \right)={\left[\frac{\partial s_{i}\left(t+\tau \right)}{\partial r_{j,k}\left(t\right)}\right]}_{i\times j\times k};\;i,k=1,\ldots ,n\;\mathrm{and}\;j=1,\ldots ,9$$

Here, i indicates the influenced cell, j the intrinsic controller node and k the influencing cell. This Jacobian tensor can be coarse-grained and visualized as a causal network that depicts how information is generated, hence organized, at the network level. A causal network of the model is defined as a directed network whose nodes represent variables (e.g., genetic states, patterning activity states, etc.) of the original model and a connection represents a non-zero influence between the connected variables. A unique causal network exists for every possible timescale τ and time t, and for every possible pair of variables X and Y (they could represent the same variable). Due to its association with arbitrary timescales τ≥1, a causal network can only be generated via mathematical integration; hence, we refer to this method as “causal network integration”. In this sense, our method could be considered as a nonlinear generalization of methods that employ network-structure-based integration techniques, such as eigenvector centrality, that implicitly assume linear dynamics [69,70]. We utilized the software package *Pytorch* [68], built on automatic differentiation techniques, to integrate the model. A discrete version of this method has been developed to integrate Boolean networks and cellular automata [71] (pp. 124–132).

The main difference between CI and most existing measures of information-processing and causality such as transfer entropy, Granger causality, etc. is that CI is a deterministic measure while the others are statistical in nature. This is because CI is computed off a directed acyclic graph (commonly referred to as DAG) representation of the operations in the dynamical model known as the “computational graph” using the chain rule [68], whereas the statistical measures utilize probability distributions that are either observed or inferred from the empirical data [72]. In other words, CI captures causality based on the linkage among the computational operations in a dynamical model, whereas the statistical measures of causality are based on empirical correlations in a dataset. A natural limitation of CI in its present form is that it requires a differentiable model and cannot be computed over data alone.

## 3. Results

We hypothesized that the biologically inspired minimal dynamical model can solve the patterning problem using self-organization alone, that is, without the aid of any external instructions or special initial conditions. We found that the model can indeed solve this problem in this way. We also found, using the causal network integration approach, that the model dynamically breaks symmetry and integrates into a macroscale network with emergent patterns whose characteristic features explain the shape of the activity pattern. Below, we describe these results in detail. We start with a description of the results of the training and the overt patterning of the best-trained model, followed by a depiction of how the patterning behavior is reflected in the activity of the internal controllers and in single-cells, concluding with a characterization of the causal network machinery that links the controller activity to the overt behavior. The results of the parallel in-depth analysis of the boundary-marker patterning are presented in SI (Figures S2–S8).

### 3.1. The Model Learns to Generate the Correct Activity Patterns and Mark Boundaries

**Figure 4:**
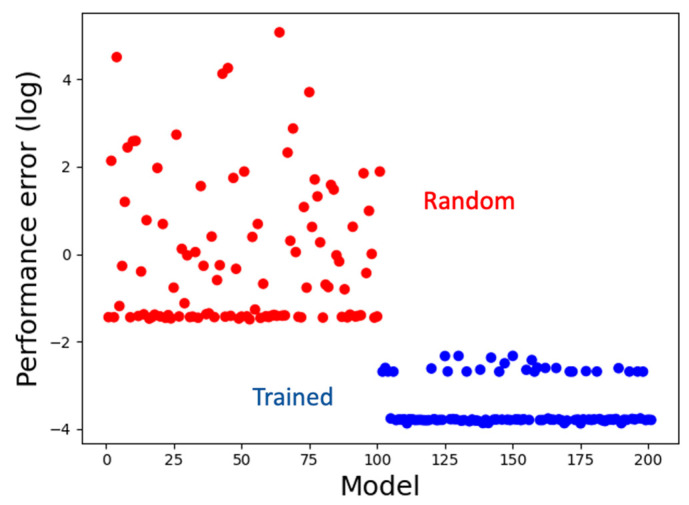
Training performance. Models trained using machine-learning have low performance errors (blue) compared to the random models (red). The line of blue dots at the bottom comprises the set of top-performing models (72% of the trained models) with similar scores.

We simultaneously trained a set of 100 models for about 100,000 iterations, a majority of which (72%) attained satisfying performance with an average MSE loss of about 0.04 and a minimum of about 0.02, compared to the performance of a set of random untrained models with an average MSE loss of about 6.1 (Figure 4), where the ideal loss is 0.0. Thus, machine-learning helped discover hypotheses (coded in the form of models) about how biological systems might solve the patterning problem. In the following sections, we analyze the best-performing model to decipher those hypotheses (information-processing strategies). We chose to analyze a single representative top-performing model because its behavior is qualitatively similar to the average behavior of the top-performing ensemble (Figure S1).

### 3.2. Analysis of Cellular Activity and Structural Patterns

#### 3.2.1. The Model Develops Network Activity and Boundary-Marker Patterns Establishing a Correct Axial Gradient Pattern within the Tissue

**Figure 5:**
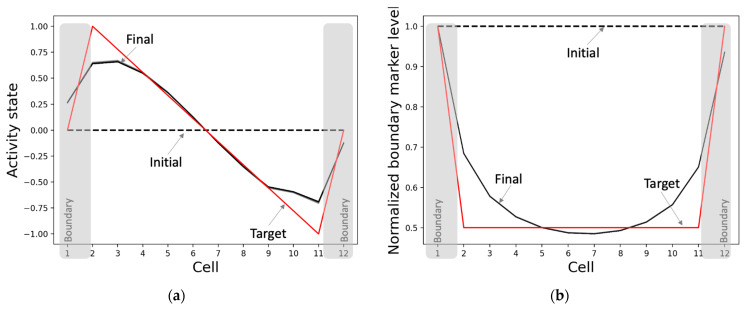
Patterning and boundary-marking behavior of the best-performing model. When initialized from homogeneous conditions and run for 4000 time-steps, the (**a**) network activity state pattern converges to a pattern (solid black) that closely matches the target pattern (red), and (**b**) the normalized boundary marker pattern reaches (solid black) a state where the cells at anterior and posterior poles have the highest levels as desired (target in red). The dashed black lines represent the initial states, and the solid black lines depict the patterns during the last 100 time-steps.

The model develops the network activity pattern and marks the boundaries starting from homogeneous conditions as expected (Figure 5). This process takes about 4000 time-steps, equaling 40 simulation-seconds, with every second comprising a sequence of 100 synchronized dynamical updates of the model’s variables. Specifically, the network develops an axial gradient pattern where the activity states drop mostly smoothly from high positive values to low negative values along the anterior-posterior axis, with the activities of the boundary cells (positions 1 and 12) tapering off towards zero (Figure 5a). In total, this constitutes a 98.4% match with the target pattern relative to the maximum mismatch of the sign-flipped target pattern. The overall (normalized) shape of the boundary-marker pattern, where the boundary cells have the highest levels of the marker and the difference between the marker levels of the adjacent cells is the largest for the boundary cells, matches the target with a score of 96.1% (Figure 5b). We also found that although the normalized pattern persists, the network-activity pattern slowly heads toward a flat shape over a long period of time. This behavior is compatible with biological examples where the positional information patterns persisting through key developmental stages disappear during the maintenance phase of adulthood or under the impact of significant aging.

#### 3.2.2. The Gap Junctions and Cell Types Also Self-Organize into Patterns Even Though They Were Not Specifically Selected for That Purpose

**Figure 6:**
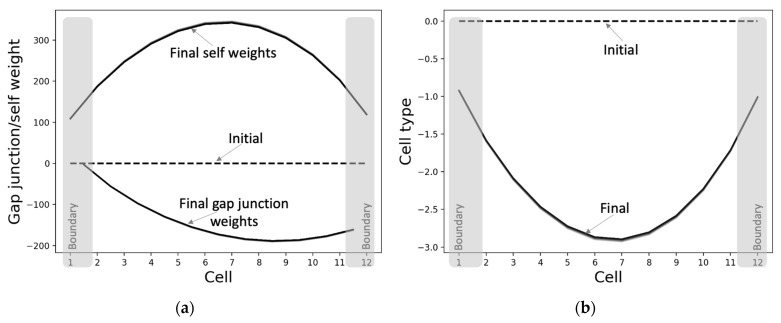
Cellular properties of the best-performing model. When initialized from homogeneous conditions and run for 4000 time-steps, the (**a**) intercellular gap-junction weights and the self-weights and (**b**) the cell types converge to characteristic shapes (black). Note that the model has eleven gap-junctions connecting the twelve cells in a chain, and every cell has a self-weight and a cell type.

The properties of the individual cells differentiate into characteristic states depending on the relative position of the cell, when initiated from homogeneous conditions (Figure 6). That is, the individual cells’ properties also assume a pattern that reflects the system-level pattern, even though no specific associated targets were provided to them during training. This suggests that patterning at the lower levels (cell properties) may be necessary for patterning at the higher levels (cell states). Furthermore, it can be observed that the asymptotic patterns of the self-weights and cell types are almost bilaterally symmetrical. This means that the cells on the opposite sides of the center behave in symmetrical ways; oppositely signed activity levels tend to change in the opposite directions (as expected). The main reason why the network-level pattern of the asymptotic gap junction weights (Figure 6a) is not bilaterally symmetrical about the center is due to the requirement that the activity states of the anterior and posterior halves should be of opposite signs; thus, the anterior weights are more positive compared to the posterior side, resulting in relatively more positive change in the activity states of the anterior compared to the posterior (Figure 3).

**Figure 7:**
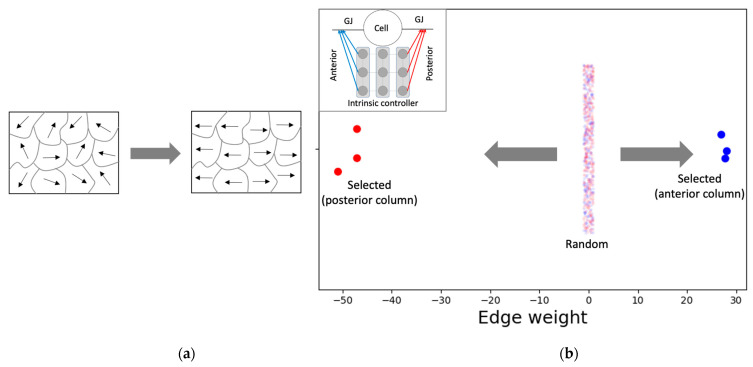
Single cells possess an intrinsic controller structure with characteristics resembling PCP. (**a**) A schematic illustrating the concept of PCP using the analogy of magnetic domains. Ferromagnetic materials contain domains within which the magnetic orientations are aligned (indicated by the arrows). The overall random pattern of orientations (left) could be modified by the application of external forces, such as magnetic fields, or temperature forcing it to assume non-random shapes (right). (**b**) Likewise, the application of a target gradient-like pattern (Figure 5) enables machine-learning to organize the initially random intrinsic controller weights into PCP-like patterns over phylogenetic timescales. Specifically, the three anterior controller weights (blue) that control the anterior GJ and the three posterior controller weights (red) that control the posterior GJ of a single cell were randomly initialized in the interval [−1, 1] during the training. At the end of training, the anterior and posterior controller weights of the representative model culminated with categorically distinct values, the anterior set positive and the posterior negative, giving the cell a character of polarity. The inset shows a blow-up of a single cell together with its intrinsic controller (Figure 2).

The PCP-like structure of a single cell (Figure 7) is an important factor that contributes to both the formation of the network-level GJ weights (Figure 6a) and the activity pattern (Figure 5a). This PCP feature is reflected in the organization of the intrinsic controller, discovered by machine-learning, with positive weights of the anterior column and negative weights of the posterior column that, respectively, control the left and right GJs of the cell (Figure 7b). This symmetry-breaking phenomenon manifests in biology in a variety of equivalent ways on an ontogenetic timescale [73] and could be considered analogous to the reorganization of magnetic domains in ferromagnetic materials due to the application of external forces (Figure 7a).

#### 3.2.3. The Model Successfully Regenerates and Rescales the Pattern despite Not Being Selected for Those Abilities

**Figure 8:**
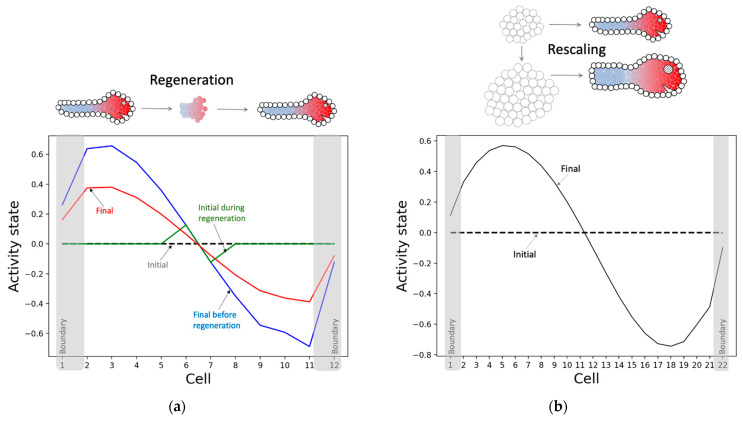
Regenerative and rescaling behaviors of the network activity. (**a**) Regeneration: the model is run for 4000 time-steps following homogeneous conditions, as before, leading to the blue pattern, then all states but that of the middle two cells are zeroed out (green) and run for another 4000 time-steps resulting in the final pattern (red). Even though the blue and red patterns do not exactly coincide they are qualitatively similar to each other. (**b**) Rescaling: the model is simulated in the same way as Figure 5a, except with 22 cells instead of the original 12 cells. With almost double the number of cells, the model takes about 3.5 times longer (14,000 time-steps) to settle, and moreover it converges (last 100 time-steps shown) to a smoother pattern compared to the 12-cell case.

The model has not only learned to solve the patterning problem it was trained for, but also to regenerate and rescale patterns—abilities that were not rewarded for during the training. Specifically, when a fully developed pattern is partly reset, where just the state of a small portion in the middle portion is retained, the model successfully regenerates the rest of the pattern (Figure 8a). Likewise, when the model is started from almost double the number of cells under homogeneous conditions as before, the model develops a larger rescaled version of the original pattern (Figure 8b). These observations suggest that the model has learnt an abstract representation of the pattern independent of the details of its generator (the model). The information-processing strategy partly underlying this ability is that almost every intrinsic controller node in every cell contains information about the network-level pattern, as described below. We hypothesize that this system-level redundancy helps the network generate the pattern regardless of the network conditions.

#### 3.2.4. The Model Generates the Same Qualitative Patterns Regardless of the Initial Network Conditions: Robustness

**Figure 9:**
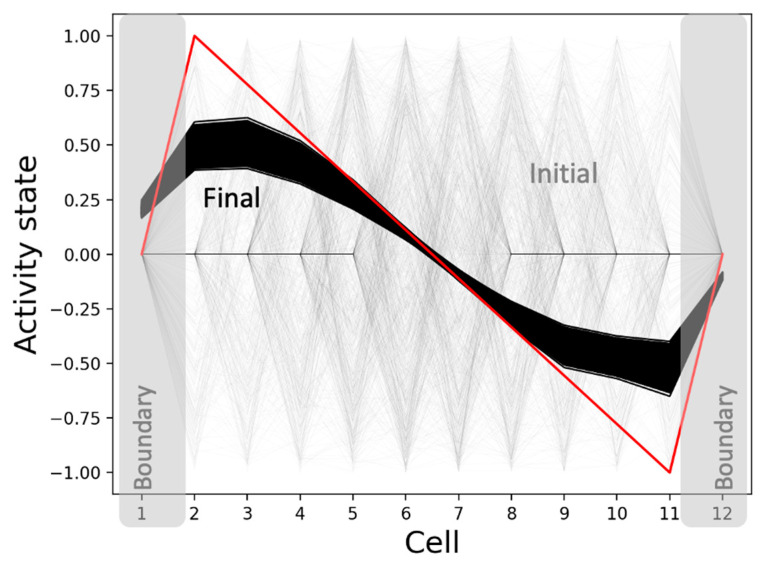
The pattern attractor-space of the activity state. The model converges to patterns (black) that are qualitatively similar to the target pattern (red) when started from a set of 1000 random initial conditions (grey). The initial conditions specifically involved a randomized initial number of ‘active’ cells whose activity states were drawn from the interval [−1, 1] and boundary-marker states from the interval [1, 2] with uniform probabilities. In the case of the ‘non-active’ cells, the activity states were set to 0 and the boundary-marker states were set to 2. The internal controller states were set to 0 in both cases.

The model is surprisingly robust to a wider variety of initial conditions (Figure 9). That is, it not only regenerates and rescales patterns under homogeneous initial conditions, but it also canalizes random initial conditions (not seen during training) into the same qualitative patterns. In other words, the model exhibits characteristics of universal robustness, which could be attributed to a distributed information-representation mechanism, also hypothesized to be responsible for regeneration and rescaling, described in more detail below. Moreover, the model also shows a slight dependency on the initial conditions (since the simulations do not converge to the exact same pattern), analogous to biological embryos typically developing phenotypes with slight variations [74,75].

### 3.3. Analysis of Intracellular Controller Activity Patterns

#### 3.3.1. Internal Controller Activity Patterns Simultaneously Correlate with Cellular Properties and Network Activity Patterns

**Figure 10:**
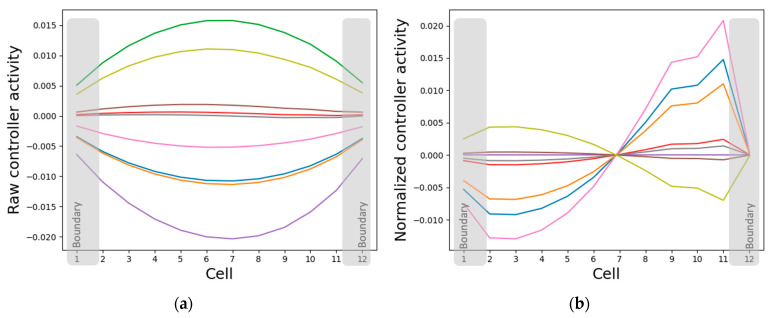
The intrinsic controller nodes’ activities simultaneously resemble the cell-properties and the network-activity patterns. Each line in the plot represents the asymptotic activity of a particular controller node across the network. That is, each line represents (**a**) the vector ($r_{i,1}\left(\tau \right),\ldots ,r_{i,n}\left(\tau \right))$ for a particular controller node i∈{1,…,9} at τ=4000 and (**b**) its cell-normalized version $\left(\hat{r_{i1}},\ldots ,\hat{r_{in}}\right)$ where $\hat{r_{ij}}=\frac{\left(r_{ij}-\underset{1\leq j\leq n}{\min}r_{ij}\right)}{\left(\underset{1\leq j\leq n}{\max}r_{ij}-\underset{1\leq j\leq n}{\min}r_{ij}\right)}$.

The states of the intrinsic controller nodes converge to patterns that simultaneously resemble the cell properties and the network activity patterns, depending on whether the states are normalized or not. Specifically, while the absolute intrinsic controller node states resemble the pattern of cellular properties (gap-junction weights, self-weights and cell types) (Figure 6 and Figure 10a), their relative states resemble the network activity pattern (Figure 5a and Figure 10b). This representation strategy makes sense since it is the same controller nodes that simultaneously influence the cell properties and are influenced by the cell state (Figure 2). Another interesting observation is that while the normalized activity state patterns of some of the intrinsic controller nodes resemble the original network activity pattern, others resemble its sign-flipped version (Figure 10b). One possible explanation for this partial inverse patterning activity is that it acts like a brake and helps the system balance the pattern, that is, it keeps the pattern from either exploding or flattening out (described in further detail in Section 3.4.2).

#### 3.3.2. Isolated Cells Contain Relevant but Insufficient Information Required to Generate the Network-Level Patterns

**Figure 11:**
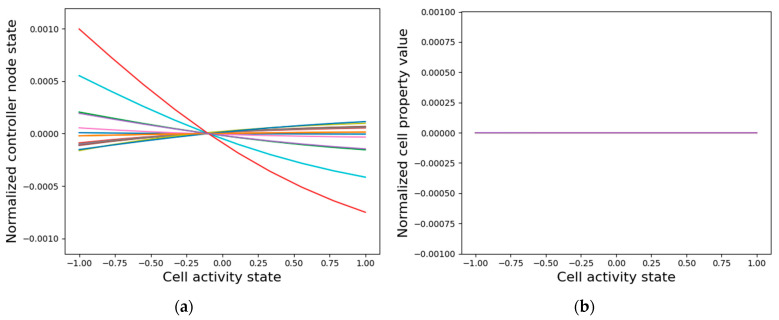
Single cells contain relevant but insufficient dynamical information about the network-level pattern. When a single cell is isolated and its external input, namely the cell activity state, is clamped and simulated for 2000 time-steps (half the time required by the network to converge), then (**a**) the internal controller nodes converge to states that clearly discriminate between the various clamped inputs. However, (**b**) the cell properties, namely the cell type, the two gap-junction weights, the self-weights converge to the same values in the respective categories regardless of the clamped input. In both cases, the states were centered at their respective mean values.

A potential explanation for why the internal controller activity patterns correlate with the cellular activity patterns, as described above, is that the controllers are inherently fine-tuned to the cell’s activity. That is, when a single cell is isolated from the network and the cell activity state is clamped, thus acting as the external input to the intrinsic controller, most controller node states tend to linearly correlate with the clamped input state (Figure 11a). However, the asymptotic GJs and cell type do not differentiate as a function of the input (Figure 11b). It is especially surprising that the GJ weights do not assume distinct values even though the anterior and posterior GJs are controlled by categorically distinct weights emanating from the intrinsic controller that give the cell a PCP-like character (Figure 7). These observations suggest that while the intrinsic controller is sensitive to single-cell activity, the cellular properties can only assume meaningful values in the collective context, a need that even the PCP-nature of the cell cannot mitigate. The learned sensitivity of the intrinsic controller is a sensible information-representation strategy, since the patterning activity states of a cell ultimately depend on the gap junction weights and the cell types that are in turn controlled by the internal signaling networks. On the other hand, it is evident that not all the information required for patterning is contained in single cells, as appropriately differentiated gap-junction weights and cell types are necessary for that purpose (Figure 6).

### 3.4. Analysis of Intercellular Causal Network Patterns

#### 3.4.1. Every Cell in the Collective Contains the Full Causal Information about the Network-Level Patterns Explaining the Model’s High Degree of Robustness

**Figure 12:**
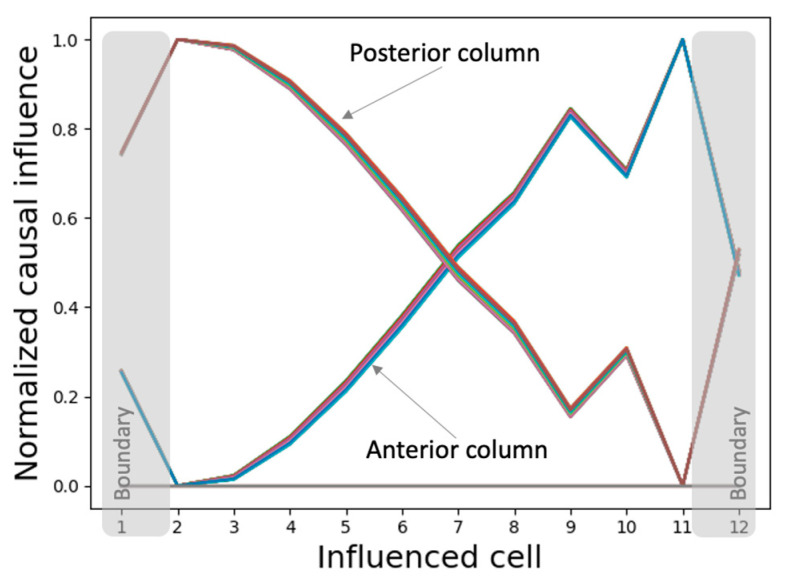
Individual nodes in the intrinsic controller network of every cell possess information about the network-level activity pattern that they control. Each line in the plot represents the normalized causal influence exerted by the initial state (t=0) of a single internal controller node in a specific cell over the asymptotic (τ=3500) activity states of the (influenced) cells, that is, it is the normalized vector $\left(\frac{\hat{\partial s_{1}\left(3500\right)}}{\partial r_{j,k}\left(0\right)},\ldots ,\frac{\hat{\partial s_{n}\left(3500\right)}}{\partial r_{j,k}\left(0\right)}\right)\mathrm{for}$ a specific controller node j∈{1,…,9} in the influencing cell k∈{1,…,n} where, $\hat{\frac{\partial s_{i}\left(\tau \right)}{\partial r_{j,k}\left(0\right)}}=\frac{\frac{\partial s_{i}\left(\tau \right)}{\partial r_{j,k}\left(0\right)}-\underset{1\leq i\leq n}{\min}\frac{\partial s_{i}\left(\tau \right)}{\partial r_{j,k}\left(0\right)}}{\underset{1\leq i\leq n}{\max}\frac{\partial s_{i}\left(\tau \right)}{\partial r_{j,k}\left(0\right)}-\underset{1\leq i\leq n}{\min}\frac{\partial s_{i}\left(\tau \right)}{\partial r_{j,k}\left(0\right)}}$.

We measured the causal influence exerted by the initial state of every internal controller node of every cell over the asymptotic network activity. The resulting patterns of causal influence (Figure 12) closely resemble the phenotypic patterns themselves, suggesting that almost every intrinsic controller node of every cell contains information about the network activity pattern in the context of the network (isolated single cells contain only partial information, as described above). This observation points to a distributed and “universal” information-representation strategy that the model employs that could also explain its ability to generate the same patterns regardless of the initial network conditions as described above. In other words, the network is tightly integrated, and the global pattern information is accessible to every gene of every cell.

Furthermore, the various spatially segregated regions (columns) of the controllers exhibit symmetrically flipped causal influence patterns, reflecting their PCP-like organization at the network level (as labelled in Figure 12). Specifically, while the posterior column of the intrinsic controller display patterns resembling the network activity pattern, the anterior column exhibits a sign-flipped version of the same. These observations could again be partly attributed to the PCP-like organization at the level of the single cell (Figure 11). As noted above, this may also be a representation of the orientational symmetry of the axis.

#### 3.4.2. The Network Dynamically Integrates into an Organization with Macro-Scale Modules Explaining the Overall Shape of the Functional Patterns

To make sense of the above results at the network level, we computed networks of causal influence between cells, where a connection from cell j to cell k represents a significant causal influence of the initial state of some internal controller node of j on the state of k at τ.

**Figure 13:**
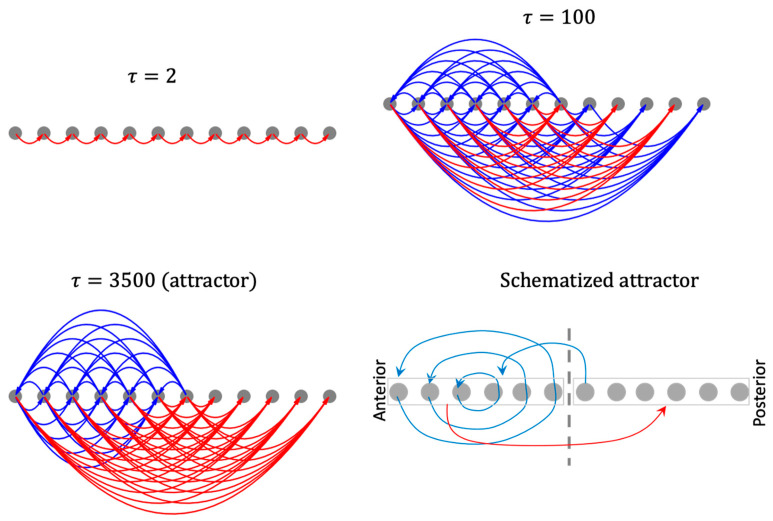
Causal network integration behind the network activity pattern developed under homogeneous initial conditions. An arrow from cell j to cell k represents the causal influence $\partial s_{k}\left(\tau \right)/\partial r_{i,j}\left(0\right)$ where $\exists i:\;\partial s_{k}\left(\tau \right)/\partial r_{i,j}\left(0\right)$ is a statistical outlier in the set $\left\{\frac{\partial s_{k}\left(\tau \right)}{\partial r_{1,j}\left(0\right)},\ldots ,\frac{\partial s_{k}\left(\tau \right)}{\partial r_{9,j}\left(0\right)}\right\}$. Blue links represent positive influence and red links represent negative influence. Multiple arrows originating from a cell may be associated with distinct intrinsic controller nodes of the originating cell.

The resulting causal networks show features characteristic of increasing complexity, symmetry-breaking, long-range influence, and the emergence of macro-scale modules with increasing timescales (Figure 13). In particular, the modular organization of the causal network attractor suggests a high-level mechanism for the gradient-shape of the asymptotic network activity pattern. For instance, while the anterior half of the network influences itself with positive feedback loops it influences the posterior half with negative influence, partly explaining why the anterior half of the activity pattern state is positive-valued while the posterior half is negative-valued. The overall mixed-feedback organization explains why the whole pattern tends to balance itself (neither flattens out nor explodes). Likewise, the causal network attractor associated with the patterning of the boundary-marker reveals an organizer-like role played by the boundary cells in that they are the only cells that influence the rest of the network (Figure S6).

This emergent modular organization cannot be explained by the structure of the original (symmetric) model itself, nor by that of the causal networks corresponding to lower timescales. In this way, the method of causal network integration partly helps close the gap between the structure and function of a complex dynamical model by focusing on the circuit-space rather than the conventional state-space.

#### 3.4.3. Rescaling the Model Rescales the Causal Networks, Explaining Why the Phenotypic Patterns Rescale

**Figure 14:**
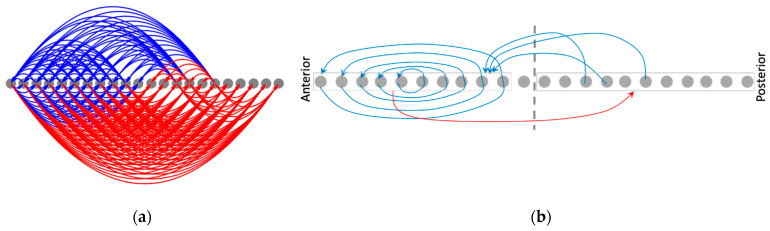
Rescaling the model (double the number of cells) rescales the corresponding causal network attractor underlying the network-activity pattern. The (**a**) causal network attractor and (**b**) its schematized version following rescaling of the model and simulating it with homogeneous initial conditions. The causal network attractor following regeneration is not shown, as it looks identical to the original (Figure 13).

The causal network integration approach offers further insights into the underlying mechanisms of patterning. It partly explains why rescaling the model (doubling the number of cells) results in the rescaling of the phenotypic patterns (Figure 8b)—the underlying causal network itself rescales (Figure 14). The macroscopic features of the rescaled causal network attractor (Figure 14b), for instance, preserve most of the modular structure of the original causal network (Figure 13), with the exception of the appearance of a couple of extra positive edges.

#### 3.4.4. The Overall Structure of the Mean Causal Network Explains the Model’s Ability to Canalize Random Initial States into the Same Patterns

**Figure 15:**
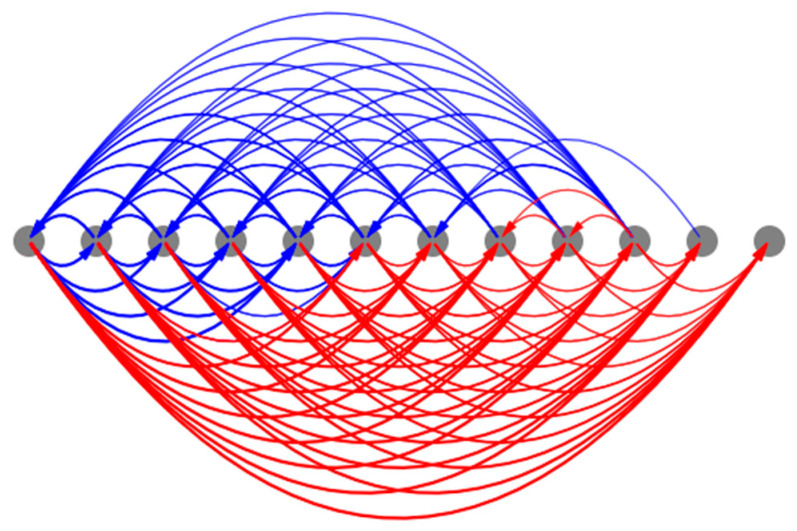
The mean causal network attractors associated with the network activity patterning. The thickness of the edges represents the frequency with which they appear in the set of attractors. The initial conditions that were used here are the same as those described in Figure 9.

One of the emergent abilities of the model is to canalize random initial states (not seen during training) to similar pattern attractors (Figure 9). The reason is that the underlying causal networks corresponding to each of those random initial conditions themselves canalize into a mean attractor causal network whose overall features match those that corresponds to the homogenous case described above. For instance, the mean causal network attractor underlying network activity patterning (Figure 15) is characterized by the same anterior-positive and posterior-negative influences as the homogeneous case (Figure 13). The reason why the causal networks themselves canalize would involve investigations that are beyond the scope of this paper.

## 4. Discussion

We have shown here that it is possible to train, using machine-learning, a recurrent self-organizing dynamical model incorporating biological priors to form gradient-like activity patterns from homogeneous conditions, that is, without the aid of externally supplied positional information or special initial conditions. We have also revealed the multi-timescale causal relationships among the components of the model, thereby describing the high-level mechanistic logic of pattern formation that the model employs.

One of the surprising findings of this work is the ability of the model to rescale the final pattern to an arbitrary number of cells (Figure 8b) despite not having been specifically trained to do so. This unexpected, emergent feature of this system mimics an interesting and important aspect of biology—plasticity. Numerous examples (for example, as reviewed in [13]) exist of robust, coherent organisms forming from the same genome despite drastic changes in the number, size, or type of cells [76,77,78,79]. The question of how certain types of search and encodings produce specifications of machinery with the ability to handle novel circumstances remains an open and important field of inquiry [80,81,82]. Our results reveal how physiological networks can embody a robust phenotypic patterning mechanism. We propose that this capability could be leveraged by evolution so that mutations resulting in an altered size of the organism need not require compensating mutations of the patterning mechanism. In other words, modularity potentiates evolution [83] by enabling a plasticity that allows organisms to maintain adaptive function (and thus fitness) while evolution explores changes in cell number.

From the perspective of morphogenesis, the causal network attractors described here could be conceived as a decoding of the “developmental program” encoded in the model’s architecture and its learned parameters. In other words, these causal networks could be understood as forms of the developmental program itself. Moreover, the same underlying model could have multiple causal networks (different projections of a single developmental program), each responsible for a unique function, such as activity-patterning or boundary-marking, as we show here. In other words, starting from the 1st order “physiological” network we have uncovered 2nd order “physiocausal” networks. Thus, our work offers new perspectives and tools to achieve one of the major goals of developmental biology—to uncover the developmental programs that organisms use for morphogenesis [1].

From the perspective of computation, the causal networks could be viewed as high-level algorithms that the underlying biophysical machinery (model) employs for the purpose of axial pattern development. By “computation” we mean information-processing or transformation of information that serves a purpose (e.g., survival or adaptive function of an organism) [84]. One might ask—isn’t the model an algorithm itself? It is what one might call a “low-level” algorithm. In a conventional algorithm (e.g., a computer program), there may be different paths that could be taken, via if-then conditions, for example. Exactly which path is taken depends on the inputs to the program. Moreover, a recurrent program that feeds the output back to itself could take different paths at different times depending on the dynamic inputs. All this suggests that even a conventional algorithm may not offer a complete explanation of the dynamic phenomenon it generates. This then raises the question: what is the ultimate high-level algorithm that describes how the given inputs are transformed into the final observed outputs in a non-recurrent and feed-forward manner? Our causal influence analysis offers a solution in this regard for our model—the causal network attractors are the high-level algorithms that offer a visual explanation for how the initial conditions are transformed into the final axial pattern. Through this analysis we also found that even though high-level algorithms, in principle, depend on initial conditions, they are all qualitatively similar to each other in our case (Figure 15). This makes sense in the context of development, as it ought to be robust enough to canalize multiple initial conditions to the same final pattern.

Viewed through the lens of the theory of computation, our model can be seen as an instance of autonomous sequential logic circuits—a type of finite state machine that does not involve external inputs. This is an appropriate class of models for developmental processes, as they are characteristically autonomous (with exceptions for environmentally triggered phenotypes, such as reviewed in [85]) and sequential at the large scale. The main limitation of this class of automata is that they are not capable of universal computation since they do not employ stacks or external tapes. At this point there is no indication that general-purpose computation is required of embryogenesis.

One of the striking features of the causal network attractors is long-distance influence: the relatively more significant asymptotic influence of the intrinsic controller of a cell on the activity state of another cell than itself, an emergent feature that is not baked in the original model (Figure 2). A biological analogue of this phenomenon would be the genes of one cell asymptotically controlling the features of another cell, that is, genetic control may not be local even though they may appear to generate only the features of the containing cell. If biological organisms indeed employ such an information-processing principle, then it would have therapeutic implications such as non-local genetic intervention. For example, might a more effective gene therapy for cancer require the hacking of the genes of neighboring healthy cells rather than themselves? Another striking feature of the causal network attractors is their modularity; cells organize into modules that tend to contain causal influences amongst themselves. Modularity is not a new concept in biology, although conventional views have focused on the overt model structure [86], and only recently has the focus widened to encompass its dynamical aspects [87,88,89]. The causal network integration approach offers a novel perspective on dynamical modularity through temporally integrated models. Such higher-order structures may lie waiting to be discovered in a variety of published biological regulatory models, containing potential high-level insights. For instance, could it be possible that the capacity of GRNs for associative memory [90] is due to a high-level causal network that is equivalent to a characteristic minimal network that is necessary and sufficient for the implementation of the memory? Overall, the tools developed here provide a new lens through which to view emergent phenomena.

Our work also offers new tools for solving the problem of top-down control in biology [13,91], where one of the open challenges is to systematically edit a complex regulatory system so that it generates a desired outcome. One way the causal network integration approach may mitigate this challenge is by offering a way to close the gap between the structure and function of a complex system by focusing on the circuit-space of the system. By offering a circuit-centric explanation of the function of a system at the top-most (asymptotic) timescale, this method thereby offers a systematic way to modify the underlying model by working the changes back down to the smallest timescale. Even though we have not worked out the details of how it could be implemented, we suspect that it would involve inferring a higher-order network model that dictates how the causal networks themselves change over time. A successful solution to the problem of top-down control would have a wide impact on biology via prescriptions for systematic interventions into biochemical networks that underlie disease. It would also impact the field of machine-learning by way of novel mechanisms of systematic supervised learning that could leverage the information contained in the macroscale structure of the causal networks.

Our work shows that the method of causal network integration has the potential to generate multi-timescale insights into how information is organized in the network. This approach of the analysis of a dynamical system focuses on the circuit-space (the space of circuits, as opposed to the space of states, induced by the model), whereas conventional approaches tend to focus on the state-space of the system. By casting the emergent dynamics at multiple scales in the circuit space, this approach brings us a step closer to closing the gap between the structure and the function of a complex dynamical system. In this regard, our method also contributes to the theory of complex systems by complementing and potentially generalizing existing approaches to characterizing canalization [88], control [92], collectivity [70,93], coarse-graining [94,95,96] and criticality in complex nonlinear dynamical systems [97,98].

All the code developed for this project can be found at: https://gitlab.com/smanicka/MinimalDevelopmentalComputation (accessed on: 4 January 2022).

## Data Availability

Data sharing not applicable.

## Supplementary Materials

The following supporting information can be downloaded at: https://www.mdpi.com/article/10.3390/e24010107/s1: Figure S1: Behavior of the best-performing model is representative of the ensemble of the top-performing models; Figure S2: Regenerative and rescaling behaviors of the boundary-marker; Figure S3: The pattern attractor-space of the boundary-marker level; Figure S4: The boundary controller nodes’ activities simultaneously resemble the boundary-marker and the network-activity patterns; Figure S5: Individual nodes in the boundary controller network of every cell possess information about the network-level boundary-marker pattern that they control; Figure S6: Causal network integration behind the boundary-marker pattern developed under homogeneous initial conditions; Figure S7: Rescaling the model (double the number of cells) rescales the corresponding causal network attractor underlying the boundary-marker pattern; Figure S8: The mean causal network attractors associated with the boundary-marker patterning.
