# Supplementary material for: Minimal Developmental Computation: A Causal Network Approach to Understand Morphogenetic Pattern Formation
Source: Entropy (Basel). 2022 Jan 10;24(1):107. doi: 10.3390/e24010107 (PMC8774453; doi:10.3390/e24010107)
Supplement: Supplementary file 1 [file entropy-24-00107-s001.zip › entropy-1392390-supplementary.pdf]

entropy

eArticle

Supplementary material

Minimal developmental computation: a causal network approach to understand morphogenetic pattern formation

Santosh Manicka and Michael Levin *

Allen Discovery Center, Tufts University, Medford, MA, USA; santosh.manicka@tufts.edu
* Correspondence: michael.levin@tufts.edu

Citation: Manicka, S.; Levin, M. Minimal Developmental Computation: A Causal Network Approach to Understand Morphogenetic Pattern Formation. *Entropy* **2022**, *24*, 107. https://doi.org/10.3390/e24010107

Academic Editors: David Wolpert and Jessica Flack

Received: 7 September 2021
Accepted: 7 January 2022
Published: 10 January 2022

Publisher's Note: MDPI stays neutral with regard to jurisdictional claims in published maps and institutional affiliations.

Copyright: © 2022 by the authors. Submitted for possible open access publication under the terms and conditions of the Creative Commons Attribution (CC BY) license (https://creativecommons.org/licenses/by/4.0/).

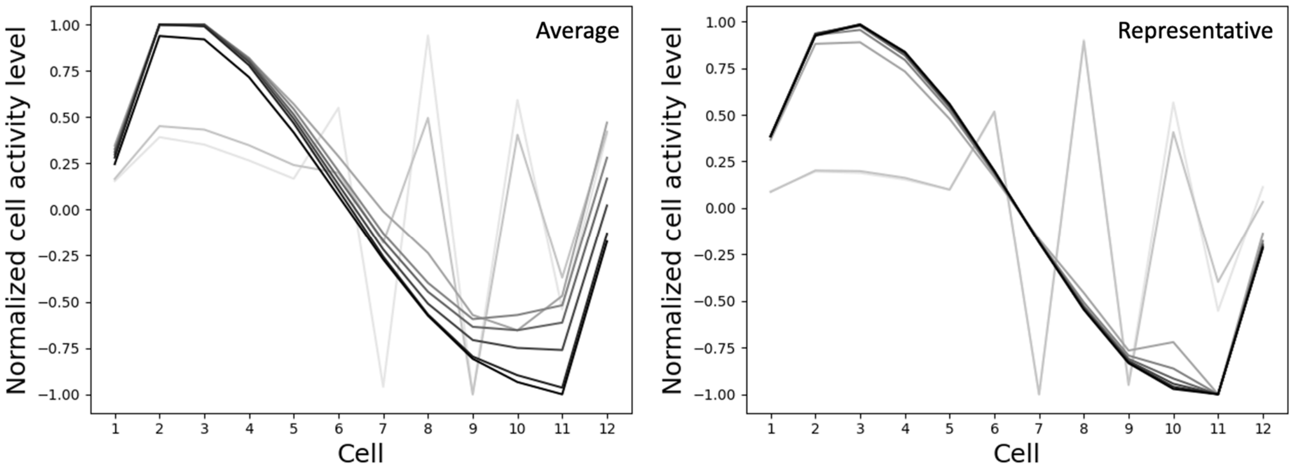

(**a**) (**b**)

Figure S1. Behavior of the best-performing model is representative of the ensemble of the top-performing models. **(a)** The average normalized cell activity levels of 72 top-performing (out of 100 trained) models depicted at the time-steps 1000, 2000, 3000, 4000, 5000, 6000, 7000 and 8000; and **(b)** the normalized cell activity levels of the representative best-performing model whose behavior is depicted at the same time-steps as (a). It can be observed that even though the two sets of behaviors don't precisely match they are qualitatively similar in that they undergo similar rugged intermediate trajectories before settling to their respective final patterns.

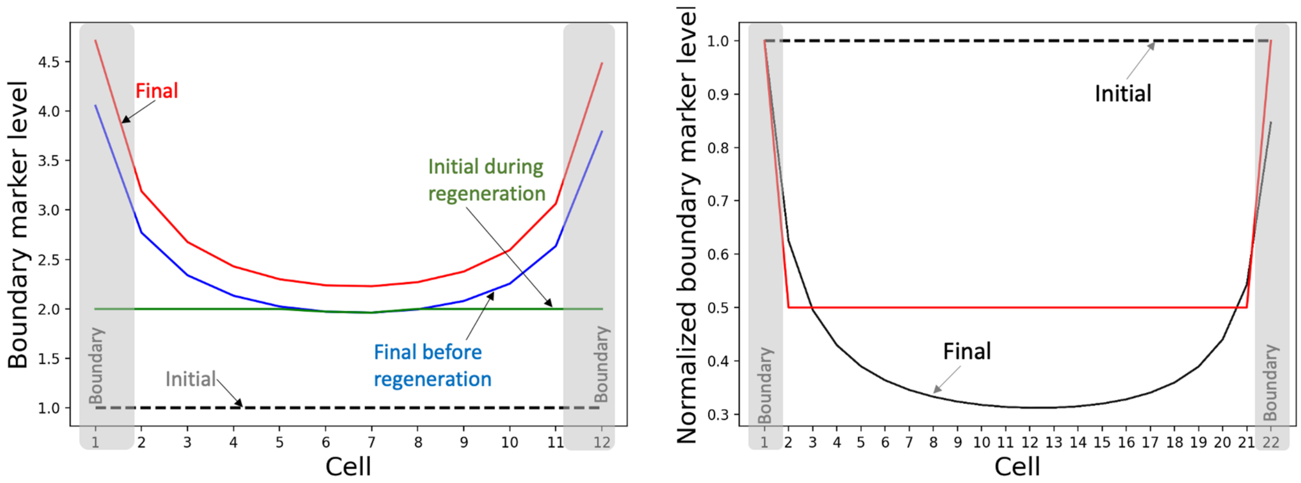

(**a**) (**b**)

Figure S2. Regenerative and rescaling behaviors of the boundary-marker. (**a**) Regeneration: the model is run for 4000 time-steps following homogeneous conditions as before leading the blue pattern, then all states but that of the middle two cells are zeroed out (green) and run for another 4000 time-steps resulting in the final pattern (red). Even though the blue and red patterns don't exactly coincide they are quite close to each other. (**b**) Rescaling: the model is simulated in the same way as Fig 5a, except with 22 cells instead of 12 cells. With almost double the number of cells, the model takes about 3.5 times longer (14000 time-steps) to settle, and moreover it converges (last 100 time-steps shown) to a smoother pattern compared to the 12-cell case.

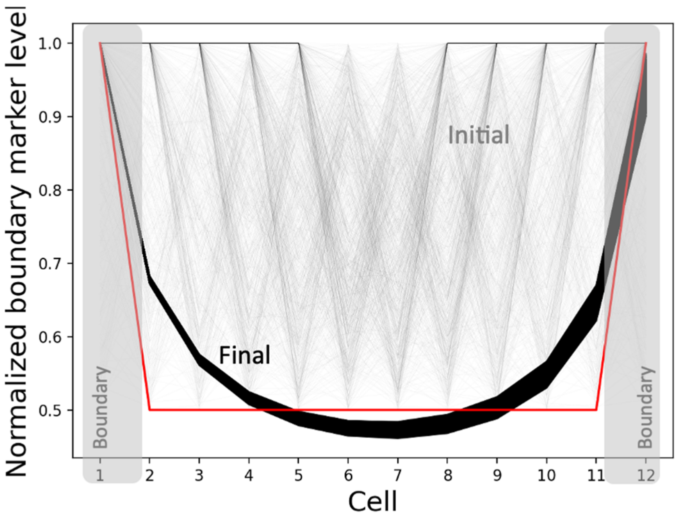

Figure S3. The pattern attractor-space of the boundary-marker level. The model converges to patterns (black) that are qualitatively similar to the target pattern (red) when started from a set of 1000 random initial conditions (grey). The initial conditions specifically involved a randomized initial number of 'active' cells whose activity states were drawn from the interval [-1,1] and boundary-marker states from the interval [1,2] with uniform probabilities. In the case of the 'non-active' cells, the activity states were set to 0 and the boundary-marker states were set to 2. The internal controller states were set to 0 in both cases.

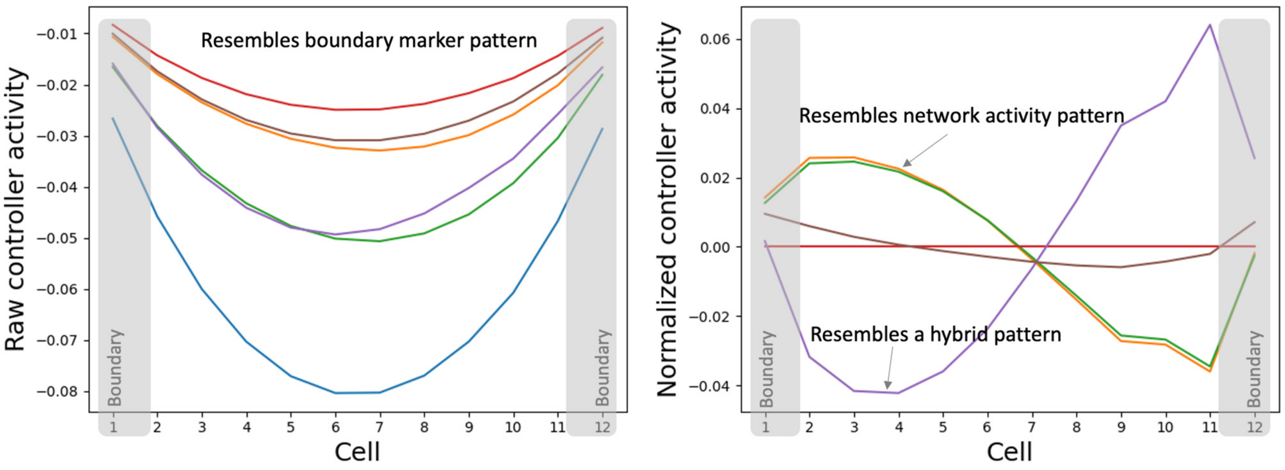

(**a**) (**b**)

Figure S4. The boundary controller nodes' activities simultaneously resemble the boundary-marker and the network-activity patterns. Each line in the plot represents the asymptotic activity of a particular controller node across the network. That is, each line represents **(a)** the vector $(y_{i,1}(\tau), \ldots, y_{i,n}(\tau))$ for a particular controller node $i \in \{1,..,6\}$ at $\tau = 4000$ and **(b)** its cell-normalized version $(\widehat{y_{11}}, \ldots, \widehat{y_{1n}})$ where $\widehat{y_{ij}} = \frac{\left(y_{ij} - \min_{1\le j\le n} y_{ij}\right)}{\left(\max_{1\le j\le n} y_{ij} - \min_{1\le j\le n} y_{ij}\right)}$. These observations could be attributed to the model design where all the nodes in the boundary controller receive inputs from both the corresponding cells' activity and boundary marker states.

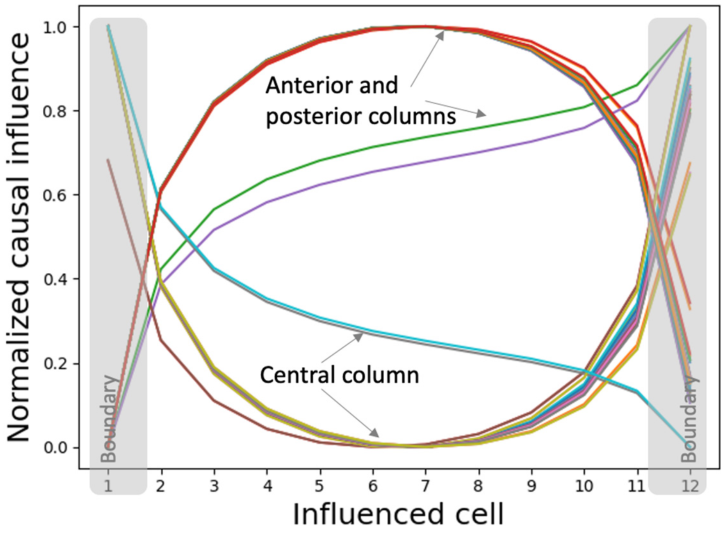

Figure S5. Individual nodes in the boundary controller network of every cell possess information about the network-level boundary-marker pattern that they control. Each line in the plot represents the normalized causal influence exerted by the initial state ($t = 0$) of a single internal controller node in a specific cell over the asymptotic ($\tau = 3500$) activity states of the (influenced) cells, that is, it's the normalized vector $\left(\frac{\partial b_1\widehat{(3500)}}{\partial y_{j,k}(0)}, \dots, \frac{\partial b\widehat{(3500)}}{\partial y_{j,k}(0)}\right)$ for a specific controller node $j \in \{1,..,6\}$ in the influencing cell $k \in \{1,..,n\}$ where, $\frac{\widehat{\partial b_i(\tau)}}{\partial y_{j,k}(0)} = \frac{\frac{\partial b_i(\tau)}{\partial y_{j,k}(0)} - \min_{1\le i\le n}\frac{\partial b_i(\tau)}{\partial y_{j,k}(0)}}{\max_{1\le i\le n}\frac{\partial b_i(\tau)}{\partial y_{j,k}(0)} - \min_{1\le i\le n}\frac{\partial b(\tau)}{\partial y_{j,k}(0)}}$.

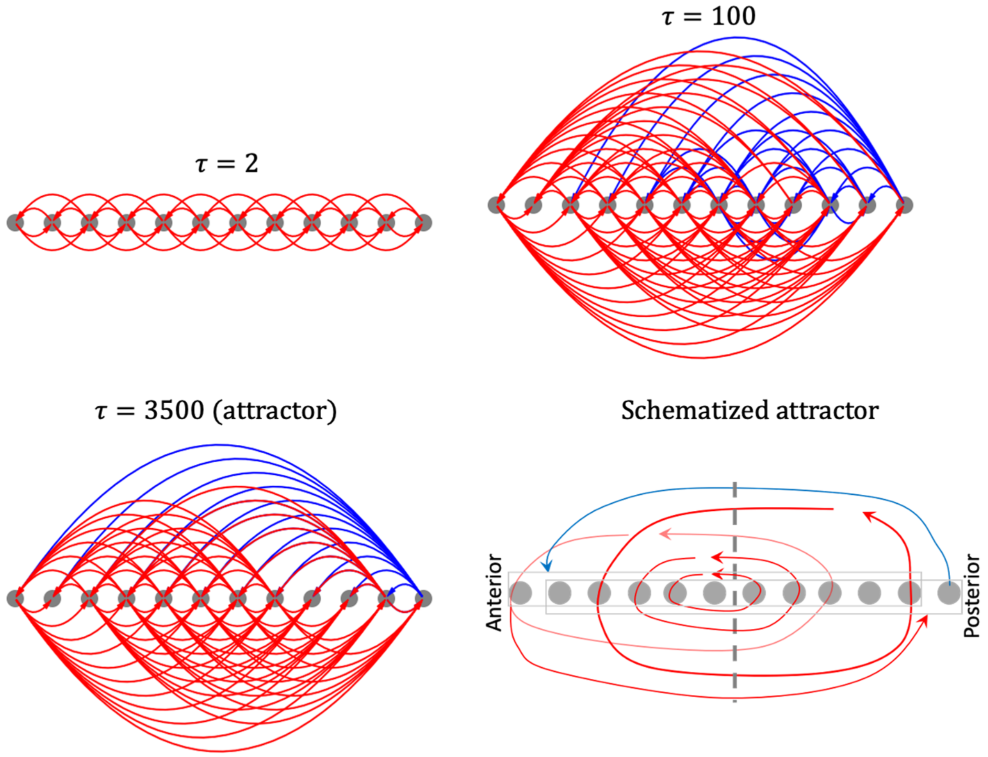

Figure S6. Causal network integration behind the boundary-marker pattern developed under homogeneous initial conditions. An arrow from cell j to cell k represents the causal influence $\partial b_k(\tau)/dy_{i,j}(0)$ where $\exists i: \partial b_k(\tau)/\partial y_{i,j}(0)$ is a statistical outlier in the set $\{\frac{\partial b_k(\tau)}{\partial y_{1,j}(0)}, \dots, \frac{\partial b_k(\tau)}{\partial y_{6,j}(0)}\}$. Blue links represent positive influence and red links represent negative influence. Multiple arrows originating from a cell may be associated with distinct intrinsic controller nodes of the originating cell.

The causal network attractor organizes into modules where most cells around the center are caught in eddies of negative feedback (one slightly more on the anterior shown in light red, and the other more on the posterior side shown in dark red) that the outer cells participate less in. Importantly, the boundary cells act as "organizers" influencing the rest of the network and each other via distinct positive and negative influences partly explaining how the model successfully marks its own boundaries. This strategy makes sense since the boundary cells are special in the sense that they have one less connection compared to the other cells, which the network capitalizes on.

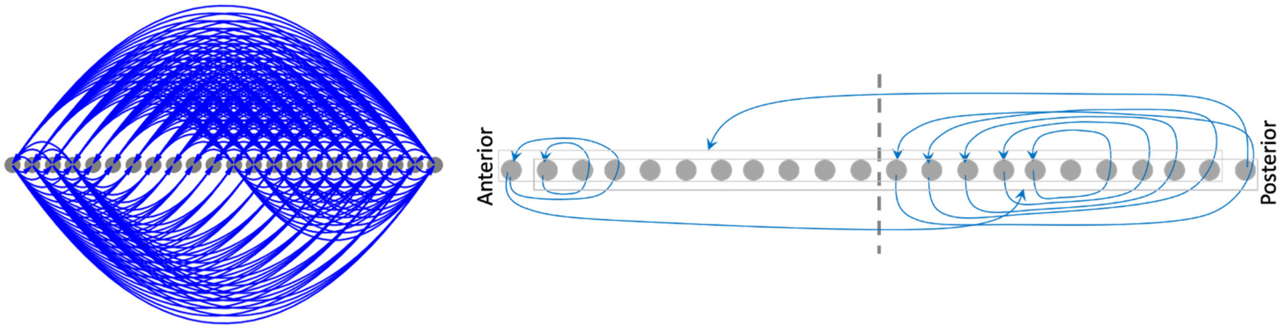

(**a**) (**b**)

Figure S7. Rescaling the model (double the number of cells) rescales the corresponding causal network attractor underlying the boundary-marker pattern. The **(a)** causal network attractor and **(b)** its schematized version following rescaling of the model and simulating it with homogeneous initial conditions. The causal network attractor following regeneration is not shown, as it looks identical to the original (Fig S6). The rescaled boundary-marker causal network preserves some but not all the characteristic features of the original (Fig S6). Specifically, although it preserves the "organizer" character of the boundary cells, it appears to have split the two eddies of the original. Moreover, the rescaled network contains only positive influences as opposed to the mixed influences of the original. An investigation of the logic behind these similarities and differences is beyond the scope of this paper.

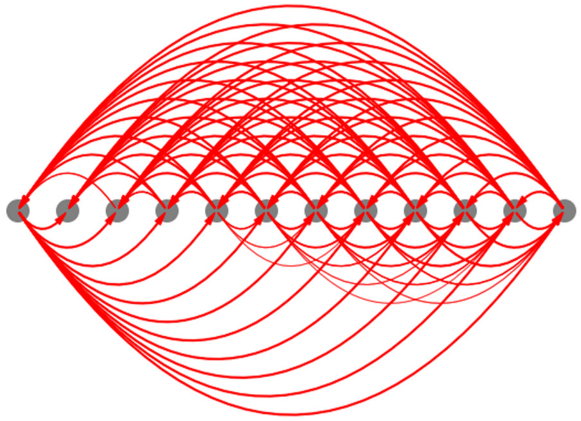

Figure S8. The mean causal network attractors associated with the boundary-marker patterning. The thickness of the edges represents the frequency with which they appear in the set of attractors. The initial conditions that were used here are the same as those described in Fig S3. This causal network attractor preserves the "organizer" character of the boundary cells as the homogeneous case (Fig S6), though it does not preserve the signs of the influences.
